# Mobile technology and cancer screening: Lessons from rural India

**DOI:** 10.7189/jogh.08.020421

**Published:** 2018-12

**Authors:** Shreya Bhatt, Rita Isaac, Madelon Finkel, Jay Evans, Liz Grant, Biswajit Paul, David Weller

**Affiliations:** 1 Medic Mobile, Mumbai, India; 2 RUHSA Department Christian Medical College, Vellore, India; 3 Weill Cornell Medical College, New York, New York, USA; 4 Centre for Population Health Sciences, University of Edinburgh, Edinburgh, UK

## Abstract

**Background:**

Rates of cervical and oral cancer in India are unacceptably high. Survival from these cancers is poor, largely due to late presentation and a lack of early diagnosis and screening programmes. Mobile Health (‘mHealth’) shows promise as a means of supporting screening activity, particularly in rural and remote communities where the required information infrastructure is lacking.

**Methods:**

We developed a mHealth prototype and ran training sessions in its use. We then implemented our mHealth-supported screening intervention in 3 sites serving poor, low-health-literacy communities: RUHSA (where cervical screening programmes were already established), Mungeli (Chhattisgarh) and Padhar (Madhya Pradesh). Screening was delivered by community health workers (CHWs – 10 from RUHSA, 8 from Mungeli and 7 from Padhar), supported by nurses (2 in Mungeli and Padhar, 5 in RUHSA): cervical screening was by VIA; oral cancer screening was by mouth inspection with illumination. Our evaluation comprised an analysis of uptake in response to screening and follow-up invitations, complemented by qualitative data from 8 key informant interviews and 2 focus groups.

**Results:**

8686 people were screened through the mHealth intervention – the majority (98%) for oral cancer. Positivity rates were 28% for cervical screening (of whom 37% attended for follow-up) and 5% for oral cancer screening (of whom 31% attended for follow-up). The mHealth prototype was very acceptable to CHWs, who felt it made the task of screening more reliable. A number of barriers to screening and follow-up in test-positive individuals were identified. Use of the mHealth prototype has had a positive effect on the social standing of the CHWs delivering the interventions.

**Conclusions:**

mHealth approaches can support cancer screening in poor rural communities with low levels of health literacy. However, they are not sufficient to overcome the range of social, cultural and financial barriers to screening and follow-up. Approaches which combine mHealth with extensive community education, tailored to levels of health literacy in the target population, and well-defined diagnostic and treatment pathways are the most likely to achieve a good response in these communities.

The burden of disease, including cancer, remains higher in low- and-middle income countries (LMICs) compared to high-income countries [1]. These global disparities in cancer outcomes arise from a range of factors including access to, and availability of cancer services, financial constraints, social and cultural factors and a lack of early diagnosis and screening services; “fragile” health care systems in LMICs limit the potential to diagnose and treat cancer effectively – especially in remote and rural regions [2,3]. A key factor in poor cancer survival outcomes in LMICs is late presentation; in the absence of early detection programmes cancers typically present at a stage where treatment may not lead to cure [4].

## Cervical and oral cancer in India

These common cancers are major causes of death in India and other LMICs - in 2012 there were 122 844 cases of cervical cancer, and 67 477 deaths; and 77 003 cases of oral cancer with 52 067 deaths [5,6]. Yet screening for cervical cancer, typically using low-technology approaches such as visual inspection with acetic acid (VIA), can be implemented effectively in LMIC settings with appropriate investment in screening infrastructure (including personnel, equipment, training) [7]. Oral cancer often arises on a background of tobacco usage (either smoking or chewing tobacco) and high alcohol intake; approximately 57% of all men and 11% of women between 15-49 years of age use some form of tobacco, and rates are higher in poor regions of India [8] Screening for oral cancer is through oral inspection by trained health care workers to detect early, treatable lesions – there is evidence of reduced mortality through oral cancer screening in high-risk populations in India [9]. Despite this evidence, there is no population-based cancer screening in India; it typically exists within small, local programmes, funded by individual hospitals, state governments, research programmes and other health agencies [10] Despite recent plans to introduce cancer screening across India [11], the majority of the population do not have access to screening services.

## mHealth and cancer screening

A key factor in implementing cancer screening in LMICs is the available information infrastructure. Successful screening requires accurate and complete recording and sharing of data on uptake, and outcomes, of screening and treatment. In environments which lack disease registers, and treatment records are poor, implementing successful screening can be challenging. Mobile Health, or “mHealth” has, in recent years, been proposed as a means of addressing the poor health care information infrastructure in many LMICs [12]. Over 97% of the world’s population has access to some form of mobile device [13] and in India about 70% of the population have a mobile phone subscription (with only minor variations between urban and rural areas) [14]. This provides the opportunity to communicate and educate, even in rural, remote areas. There is growing evidence that mHealth approaches can provide a platform of implementing health initiatives, in areas such as diabetes and health, in LMICs [15]. They’ve achieved this through a range of strategies including improved health communication, education of target populations and recording of health data. To date, however, there is little evidence on the effectiveness of mHealth approaches in cancer screening in LMICs.

## CHW-delivered cancer screening in Tamil Nadu

For over a decade Christian Medical College, has delivered a programme of cervical screening to a poor and rural population, through its outreach health service RUHSA (Rural Unit for Health and Social Affairs). RUHSA was created in 1977 to serve a rural community in ‘KV Kuppam block’, Vellore district to develop a model rural health care centre promoting health through provision of affordable medical care, innovations in rural development and through it contribute to alleviation of poverty. The programme trains community health workers (CHWs), drawn from local village communities, to provide basic health services and community-based health education; innovative techniques such as puppet theatres are used to deliver health prevention messages tailored to low-health-literacy populations [16]. Their cervical screening programme uses low-technology screen-and-treat approaches, and has achieved excellent levels of uptake – nevertheless, they’ve encountered significant socio-cultural barriers to gaining trust and acceptance among the local population, and loss to follow up among test-positive women (due to issues including fear of treatment and cost) has been an on-going issue [17].

To improve screening delivery and outcomes in these underserved populations, there is potential to combine CHW-delivered screening interventions with mHealth approaches. We report on a project in 3 rural and remote regions of India (including RUHSA) in which we developed a “mHealth prototype” and used it as a key component of screening programmes for cervical and oral cancer. Our objectives were to determine: 1) the key features of an ideal mHealth prototype for use in cancer screening in LMIC settings; 2) the views of CHWs, nurses and others involved in the delivery of the programme, on feasibility of using the prototype, how acceptable they found it, and how it might be improved; and 3) the response of the target population to screening invitations from the programme (detailed data on screening rates and outcomes, before and after our intervention, are reported elsewhere). The project was undertaken between April 2016 and March 2018.

## METHODS

The study sites are described in **Table 1**. They comprised three mission hospitals (Christian Medical College Vellore’s Rural Unit for Health and Social Affairs (RUHSA) in Tamil Nadu; Padhar Hospital in Madhya Pradesh; and Christian Hospital Mungeli in Chhattisgarh). They were selected for the following characteristics:

**Table 1:** Study sites

Site
**Rhusa:**
Access to diagnostic and treatment facilities of Christian Medical College
Has been providing community outreach and cancer screening for over a decade with great success
Uses CHWs (also known as health aides); local women hired and trained to do community health outreach – services include building educational awareness ND providing antenatal care at the sub-centers
Provides primary and secondary health care through its 75-bed community health center and 18 sub-centers to a population of approximately 120 000
**Padhar Hospital:**
Established in 1958, a 200-bed, multi-specialty hospital with a relatively new community health program, providing care to the surrounding tribal villages [26]
Also relies on paid CHWs to provide care services as well as non-health social support services through a combination of household visits and at organized health camps.
**Christian Hospital Mungeli:**
Established in 1896, a 120-bed rural hospital providing a wide range of medical services to the surrounding communities [27]
Access to a low-cost cancer radiation and chemotherapy services
No CHW program - this project, Mungeli recruited nursing students (known as community college nursing assistants or CCNAs) and train them as CHWs

CHW – community health worker

- provided care to underserved, very low-income populations,
- were the only hospitals in their respective vicinity,
- served populations with a high incidence of late-stage cervical and oral cancers,
- had oncology and radiology departments that can provide advanced cancer treatment, and
- hospital directors agreed to participate in the project.

### Development of the mHealth prototype

The mHealth prototype was develop by Medic Mobile, a non-profit technology organization that designs, builds and implements open-source software for community health workers, including a parallel low-cost SIM card application operating on thin SIM cards [20]. The prototype was based on “human-centered design” [21]; this approach prioritises the needs of the users of the prototype, and takes into account the context and setting in which it will be used. It also promotes user engagement with the technology, and a sense of “ownership” [22]. Key elements of the prototype development process are shown in **Table 2**.

**Table 2:** The mHealth prototype development process

• Medic Mobile team visited each site to get a sense of current cancer screening activities and their strengths and weaknesses, the effectiveness of the activities and the challenges faced
• Through in-depth interviews, role playing, and shadowing, the team gained an understanding of the workflow for screening activities – and defined ‘user personas’ (a representation of who a user is, and what their behaviours are) – for CHWs, nurses, and program coordinators)
• Assessed the feasibility of using mobile technology within the existing infrastructure (physical and personnel), taking into account user-related considerations
• Assessment of existing mobile networks at each site and extent of mobile phone ownership
• Feature phones loaded with a configured SIM card application and provided to the chws at each site
• Conducted mHealth training workshops
• Prototype tested at a pilot ‘screening camp’ to test the platform in a ‘real world’ setting, and gauge training gaps among the staff
• SIM application configured to support specific data collection forms, allowing users to report on each type of activity

CHW – community health worker

Medic Mobile’s prototype was tailored to meet site characteristics (for example, CHWs at RUHSA had higher qualifications and experience than in the other settings). Further, while all of the hospitals had internet coverage, there were discrepancies in mobile internet coverage in the local communities, (especially in the hill tribal communities). This meant that the intervention had to be able to function offline.

### Key features of the mHealth prototype

The SIM application we used was configured to support specific data collection forms, allowing users to report on each type of activity: two separate forms for cervical cancer and oral cancer screening, and a form for confirmation of treatment for both cancers. The screening form collected patient demographic details as well as a range of symptom-related questions for cervical cancer and behavior-related questions for oral cancer. The other two forms recorded whether or not a second test, a biopsy, and/or treatment was completed, and included the results for each.

All data needed to be manually entered on the prototype, and security and permissions were set at levels to minimize the risks of personal identifying data security breaches. There were no social media linkages – it was a basic feature phone with limited user interactivity, with data transmission by standard SMS messaging.

For the screening workflows, the nurses received a five-digit unique identification number for the patient and data were entered onto a patient e-record. While CHWs typically owned mobile phones, they were supplied with simple feature phones for this project (based on levels of health literacy and ease of use). The SIM card application loaded on the phone was menu-driven, and facilitated the collection and transmission of large quantities of data. This decision not only suited the constraints of limited internet connectivity in the local communities, but also aligned with user preferences, literacy, and comfort levels.

Pilot-testing enabled the study investigators to test the workflow and clinical protocols, and monitor for gaps in community awareness-building, and patient management. Most importantly, it provided users with a sense of confidence in the ability to perform the tasks required of them. Once the prototype was in use, we established a feedback loop to report on technical and other issues in its use; a number of refinements were made over the course of the evaluation. Feedback on the technical and personnel constraints experienced during the course of the project led to continuous refinement of the technology, as well as workflows.

### The screening process

Trained nurses conducted the cervical cancer screening at the village satellite clinics or in common community buildings using visual inspection with acetic acid (VIA). Test-positive individuals were invited to attend the local gynaecology clinic at secondary level care health centers for confirmatory investigations (further VIA and biopsy) and, if necessary treatment. For the oral cancer screening CHWs undertook oral cavity inspection; patients with precancerous lesions were referred to the dental surgeons in the respective health centers for further testing and treatment.

The mHealth prototype was used to provide a five-digit unique identification number for each patient and register patients into an appropriate screening and referral workflow. All screening, follow up results and treatment outcomes for registered patients were subsequently recorded on the prototype. In this way, the programme coordinators were able to monitor screening participation, loss to follow up, and treatment outcomes; they used this information to coordinate efforts to undertake further screening and follow-up of non-responders to confirmatory diagnostic tests

### Training in use of mHealth prototype

A number of training workshops were held at each of the study sites. At the Padhar and Mungeli sites, the training included sessions on community awareness-building and educating the public, emphasizing the importance of health preventive activities (this was less-emphasised at RUHSA where community education was already well-established), and on clinical protocols for screening and follow-up of cervical and oral cancer. All workshops were attended by nurses, the CHWs, programme coordinators, and clinicians providing gynaecological and dentistry/oral surgery services.

The workshops the use of our mHealth prototype with its associated SIM application for CHWs and nurses - as well as the linked web application for the programme coordinators receiving the data. At each of the three sites, CHWs and nurses attended 3-4 hour sessions on the use of the mHealth tool and were trained using a range of methods including demonstrations, role-playing, group activities and practice exercises. Each workshop provided the staff with training materials, including a comprehensive guide for programme coordinators and a step-by-step tool guide for the CHWs and nurses. A pre-test/post-test training assessment was conducted at each site and demonstrated that, at the conclusion of the training, the trained staff were comfortable with the use of the feature phone.

### Interviews

Once screening had been undertaken for at least six months, we conducted 2 focus group discussions (each with 6 participants) and 8 key informant interviews with CHWs and programme coordinators. This qualitative data collection as limited to the Padhar and Mungeli sites where, in contrast to RUHSA, screening was a new innovation. We sought a representative sample of participants - our schedule of group and interview discussions is shown in **Table 3**. The interviews were semi-structured, with a standardized pro-forma, and sought descriptions of participants’ experiences with the mHealth prototype, and their impressions of whether it made the various tasks of screening (including identification of invitees, recording of information and results and follow-up of test-positive cases) easier – when compared with standard paper-based techniques. There were also questions on reported confidence in using the prototype, and any perceived changes in the way they were treated by people in the families and community since being given the prototype. All 15 CHWs from Mungeli and Padhar were invited, and 10 participated in either a key informant interview or focus group. The remaining 10 participants comprised nurses, programme coordinators and other administrative personnel involved in the delivery of the programmes

**Table 3:** Description of focus group and key informant interviews

	Sites and format of interviews	Participant characteristics
**Padhar**	The discussion was conducted in Padhar Hospital’s community team office room. Participants were given a brief introduction to the discussion format and ground rules, and were then asked questions across several themes. Follow-up questions were used to elicit further detail when appropriate. Detailed responses were noted down and later transcribed.	From Padhar Hospital’s community outreach team, 2 nurses & 5 health workers using the Medic Mobile application, and 1 Program Administrator participated in the discussion.
**Mungeli**	The discussion was conducted in Mungeli’s mobile clinic unit. Participants were given a brief introduction to the discussion format and ground rules, and were then asked questions across several themes. Follow-up questions were used to elicit further detail when appropriate. Detailed responses were noted down and later transcribed.	From Mungeli’s mobile unit team, 2 nursing assistants using the Medic Mobile application participated in the discussion.

The discussions took place at a variety of venues, including hospitals and in mobile outreach units. Data from the interviews were transcribed, and underwent a thematic analysis, using a grounded theory approach; we applied an inductive process whereby theoretical insights were generated from our data [23]. In brief, we first became familiar with the interview and focus group data, looking for initial patterns and themes, leading to the generation of our initial codes. We then searched more thoroughly for themes, focusing particularly on issues of usability, acceptability and perceived utility. These themes were then further developed and refined, with the aim of grounding our findings in the realities of the participants as they worked on this project.

### Ethical approval

We received ethical approval from the Institutional Research Board of Christian Medical College, Vellore

## RESULTS

### Uptake and usage of the mHealth prototype

Mean follow up of the 34 prototype users (25 CHWs, 9 nurses) was 14 months from the time they received the feature phone and began using it. CHWs and nurses at each site continued, over the course of our evaluation, to actively using the mHealth prototype to report screening, test results, and treatment. A total of 8686 screening forms were submitted using the mHealth tool over the 23-month evaluation period; 2% (n = 170) were cervical cancer screenings, the remainder were for oral screening.

### Impact on follow-up

Of the 170 women who were screened for cervical cancer, 49 (28%) tested positive on VIA. Of these 18 (36.7%) attended the hospital/clinic for follow-up testing and/or biopsy despite continuous follow-up by health workers. Amongst those (8,516) who were screened for oral cancer, 5% (n = 490) tested positive, but only 151 (30.8%) attended for follow-up.

### Results of interviews and focus groups

#### Perceived ease of use

Most participants felt the prototype was simple and easy to use; they typically indicated that the prototype promoted efficiency in a variety of ways, including reduced reporting time, less data errors, and enablement of faster case management and follow up in real-time. Younger CHWs and programme coordinators however, expressed a preference for smartphones over feature phones.

CHW, Padhar: “Since it’s step-by-step on the phones, it’s easy to ask all of the questions step-by-step using the phone. We can double-check each question before sending (if we’ve made any mistakes or left any questions blank), we revise all questions before sending. We get the patient ID SMSs immediately”

#### Perceived impact and social benefits

Most participants reported that the mHealth prototype had a psychological impact effect on them – typically they felt that being provided with a phone, and the associated training, led to an increase in self-confidence, self-esteem, and motivation. Female staff, in particular, typically reported a greater sense of confidence in their own abilities. Although the use of the mHealth tool was new to them, especially at Padhar and Mungeli, participants were impressed by how the tool supported real-time communication - and how it helped them to coordinate care of their patients for follow-ups and referrals. They also reported positive comments from their patients, who often viewed the mobile tools as a commitment of the hospital to their care and well-being. Despite greater coordination of care, however, it is important to note that further investigation and treatment rates for test-positive cases were significantly low given a range of socio-economic barriers to treatment.

Community College Nurse assistant, Mungeli: “Our families like it and are happy that we use phones for our work. My husband says ‘at least now you are doing something’. He proudly tells others that I got a phone from the hospital.”

Nurse, Padhar: “We feel proud [to use mobile phones], it’s a new way [of doing our work]. Even others must be impressed that we’re doing it [our work] on the phone.”

#### Perceived advantages over paper-based information sharing

Health workers reported that the mobile tools made reporting much easier and quicker as compared to paper-based systems which were more cumbersome to carry out in the field and time-consuming to maintain.

CHW, Padhar: “The phones have helped us become quicker and more efficient. Using paper forms [for reporting] would have been so time-consuming. I also now have greater self-confidence that I’m able to do this well”

#### Reported barriers to screening

The CHWs and nurses were typically immersed in the communities involved in the programme. They reported significant barriers to uptake of screening, including social and cultural factors and financial constraints. Several respondents raised the issue of time off work; cost of transportation and losing a day’s wages to attend a hospital OPD appointment that pose a very significant challenge for patients.

Supervisor, Padhar: “The woman class in rural setting don’t have any money. Money is controlled by husbands. Even if they [the women] are willing to come [for treatment], they can't come. This is the hard truth.”

#### Effect of the prototype on follow-up

The CHWs and nurses were disheartened by the impact of the mHealth intervention on take-up of follow-up investigations amongst test-positive individuals.

Nurse, Padhar: “We found a few positive cases in cervical cancer, but after screening, getting them to hospital is difficult. Even if we give them education about the seriousness of the condition, most people don’t have money for the biopsy, treatment etc.”

#### Differences between user groups

We were unable to detect any clear differences in responses between the CHWs and nurses, despite differences in educational background and training.

## DISCUSSION

Our pilot mHealth project, conducted in rural, poor regions of India, showed that a simple, low-cost mHealth tool can achieve high levels of uptake and usage amongst a group of trained CHWs and nurses. Despite some of the challenges with the technology, logistics, and socio-cultural behaviors, the mHealth tool was considered, by users, to have improved health workers’ efficiency and increased staff motivation. Importantly, our results demonstrate that mHealth interventions work best when they are introduced into an environment where there are existing well-defined pathways for diagnosis and treatment (such as RUHSA). They also need complementary programs such as community health education, and well-trained ancillary staff.

The prototype’s perceived ease of use is encouraging; our users were working in challenging settings (including variable mobile phone coverage, frequently very busy clinics etc) but still identified advantages over paper-based strategies. The social impact findings were interesting – we didn’t fully anticipate the extent to which provision of the mHealth prototype to CHWs would alter their social standing in their communities and families – this a potential added effect of our intervention. Indeed, mHealth approaches may have an ‘empowering’ effect on health care workers in poor, rural settings which may, in turn, motivate them to perform their preventive activities to a higher level - it’s an area which warrants further exploration.

Technical issues with both the hardware and the onsite infrastructure were encountered at various points during the pilot program, requiring refinement of the prototype. For example, the SIM applications were cumbersome to manage; any changes to the various forms used to collect the data required that all project SIM cards had to be collected and updated by the project team. Breakage and general wear-and-tear posed an additional hardware challenge. Replacement SIM cards, unavailable at each site, had to be couriered to the sites. A major technical challenges was unstable mobile connectivity. In the hillier regions and in communities with limited or poor cellular coverage the CHWs and program coordinators faced significant delays in receiving patient unique IDs as well as acknowledgement of screening report submissions.

We had hoped that use of the mHealth prototype would improve follow-up in test-positive screenees; better recording and transmission of data might, in theory, improve a screening programme’s ability to keep track of screen-positive individuals, and persuade them to participate in diagnostic tests after their test results. This wasn’t the case; indeed, RUHSA, there was no improvement from previous rates of follow-up. We conclude that mHealth approaches, with their more efficient delivery of information to CHWs and screenees isn’t, in itself, sufficient to address this significant issue – indeed, as other studies have shown, these approaches need to be integrated with multi-faceted programmes designed to overcome social, financial and cultural barriers for full participation in preventive health activities [24,25].

Despite the disappointing impact on follow-up investigations, CHWs and nurses were positive about the prototype’s ability to provide ‘real-time’ information at the point of delivery of screening services – which they felt had great potential to better coordinate screening activities. Further work is needed to explore mHealth approaches in populations which have had limited exposure to health education and preventive activities. Ideally, integrated approaches, in which mHealth interventions are delivered in the context of community and health worker education programmes, and other strategies to overcome barriers to screening participation, should be evaluated. Our results suggest that it is possible to build a low-cost mHealth prototype with the capacity to collect, store and transmit data, which is acceptable and considered helpful by CHWs in low-resource settings. An ideal prototype might build on this with some ‘smartphone’ features, allowing, for example, transmission of images and videos (to assist in diagnosis and conveying educational/health promotion messages). However, access to wi-fi technology, and affordability of smartphones with their associated with 3G/4G cellular networks charges places limits on their use – indeed, a focus on the development of low-cost audible and readable message delivery (such as we used) has been widely advocated in poor and rural settings [26]. mHealth strategies which might overcome system, financial and socio-cultural barriers to preventive health are being actively sought by governments in LMICs [27], and are even seen as way of helping achieve the goal of universal health coverage. Our paper adds to a growing body of evidence that the technology of mHealth must be matched by the resources and care pathways in target communities if it is to help produce favourable health outcomes.

## CONCLUSION

There are many challenges in introducing a screening-related mHealth intervention in low-health-literacy communities, but mHealth prototypes can work well in such settings. mHealth tools can increase health worker efficiency and improve care coordination between patient and provider. They work best when embedded into an ecosystem with established pathways to care so that patients have access to timely and affordable preventive care, and to appropriate investigations and treatment. Education and promotion of cancer awareness are vital accompaniments to future mHealth strategies targeting cancer screening and early diagnosis in low-income countries.
